# Traditional Dietary Recommendations for the Prevention of Cardiovascular Disease: Do They Meet the Needs of Our Patients?

**DOI:** 10.1155/2012/367898

**Published:** 2012-02-28

**Authors:** Johannes Scholl

**Affiliations:** Prevention First, 65385 Ruedesheim, Germany

## Abstract

The characteristics of patients with CVD have changed: whereas smoking prevalence declines, obesity and metabolic syndrome are on the rise. Unfortunately, the traditional *low-fat diet *for the prevention of cardiovascular disease (CVD) still seems to be the “mainstream knowledge” despite contradicting evidence. But lowering LDL-cholesterol by the wrong diet even may be counterproductive, if sd-LDL is raised and HDL is lowered. New insights into the pathophysiology of insulin resistance and its influence on the effects of dietary changes have led to a better approach: (1) the higher a patient's insulin resistance, the more important is the glycemic load of the diet. (2) Fat quality is much more important than fat quantity. (3) The best principle for a reduced calorie intake is not fat counting, but a high volume diet with low energy density, which means fibre rich vegetables and fruits. (4) And finally, satiation and palatability of a diet is very important: there is no success without the patient's compliance. Thus, the best approach to the dietary prevention of CVD is a Mediterranean style low-carb diet represented in the LOGI pyramid. Dietary guidelines for the prevention of CVD should to be revised accordingly.

## 1. Introduction

In nutritional medicine evidence is more difficult to obtain than in cardiovascular drug studies, where RCTs with hard end-points are the gold standard. Conclusions in nutritional medicine are frequently drawn from epidemiological studies, which are subject to many possible confounders. There are very few randomised studies with coronary heart disease (CHD) or total mortality as the endpoint, and those studies (like some trials in mental hospitals in the 1970s) do not meet our current ethical and scientific standards. Unlike laboratory animals, human beings cannot be fed over a long time; thus most nutritional intervention studies are short to medium term and focus on surrogate parameters. 

Controlled dietary intervention studies nevertheless can give important insights into the possibilities of dietary changes. If there are known pathophysiological mechanisms, a dose-response relation in the intervention, biological plausibility, and consistency with epidemiological studies, then effects of dietary changes can be considered as plausible.

One big misconception should be avoided: hardly any food is “bad” or “good” for health *per se*. When people eat less of one kind of food, they will likely exchange it for a different one, because they usually eat “ad libitum”, meaning until they are satisfied. So it is always about exchanging one food item for another one—and the main question is, which changes are best for our patient's health.

The intention of this paper is to stimulate discussions about whether the current guidelines should be adapted to the latest evidence and to give an insight into the ongoing debate, which diets are best for the prevention of cardiovascular disease.

## 2. Diet and Heart Disease

Traditionally, low-cholesterol (LC) and low-fat-high-carb (LFHC) diets have been recommended with the intention to reduce cardiovascular disease (CVD) and obesity risk. The evidence behind this dietary strategy is questionable.

Recent data from NHANES show trends in dietary macronutrient composition, total energy intake, and the prevalence of overweight and obesity from 1971 to 2006. While relative percentage of fat in the diet decreased significantly from 36,6% to 33,7% and the percentage of carbohydrates increased from 44,0 to 48,7%, which complied with the official LFHC recommendations, total energy intake increased, too, and US people became fatter and fatter [2]. This casts doubt on whether the recommendation to “eat less fat” has had any beneficial effect on a population level.

A recent “systematic review of the evidence supporting a causal link between dietary factors and coronary heart disease” came to the conclusion that strong evidence supported a protective associations between vegetables, nuts, monounsaturated fatty acids and a “mediterranean” style, high-quality diet and coronary heart disease (CHD), and harmful associations with the intake of transfatty acids and foods with a high glycemic index or load. Neither for saturated fat nor for eggs, there was sufficient evidence for any harm [3].

For more than ten years, leading experts in the field have been advocating a paradigm shift in dietary recommendations for the prevention of heart disease [1, 4, 5]. They are opposed by strong lobbyism of the food industry, who is against any regulations, for example, of sugar-sweetened beverages or labelling of processed food with high energy density.

In routine, daily patient care, doctors still take remarkably little notice of this controversy and of the new, high-quality evidence about the effects of dietary changes, which has been accumulated within the last decade.

## 3. Changing Patients, Changing Recommendations?

The “typical patient” with CHD may have changed in the last decades. Smoking prevalence has declined whereas overweight and obesity, Metabolic Syndrome (MetS), and Type 2 diabetes mellitus (T2DM) are on the rise.

About two-thirds of patients with coronary heart disease have either T2DM or an impaired glucose tolerance (IGT) [6]. The increasing prevalence of the MetS and the increasing incidence of T2DM in industrialized countries may lead in a long-term perspective to a greater lifetime burden of cardiovascular disease.

Recommendations must meet the needs of the target population and must thus be adapted to their metabolic profile. Within the last 10–15 years, high-quality evidence has been accumulated through dietary intervention studies in dyslipidemic, prediabetic, and/or diabetic subjects, in lean and in obese subjects with and without insulin resistance, which have clarified the influence of dietary changes on lipids and apolipoproteins, weight reduction, glycaemia, and insulinemia. It is important to incorporate this new evidence into dietary guidelines for cardiovascular prevention.

## 4. Who Is the Target Population?

The *target population* is individuals with

- the metabolic syndrome (MS) and insulin resistance,
- hypertension,
- hypercholesterolemia,
- overweight and obesity,

and very often combinations of the four.

Patients with MS have a twofold increased risk for CVD already before the manifestation of T2DM and are typically characterized by the (pre)diabetic dyslipoproteinemia (small-dense LDL, high ApoB, low HDL, low Apo-A1, high triglycerides) and fasting as well as postprandial hyperinsulinemia, in many cases by arterial hypertension and signs of premature atherosclerosis (raised IMT), too.

## 5. What Are the Objectives of Dietary Changes in Target Population?

The main objective is to lower the individuals' overall cardiovascular disease (CVD) risk. Dietary recommendations for this target population should therefore

- improve dyslipoproteinemia (lower TG, increase HDL, lower TC : HDL ratio, lower small dense LDL, and ApoB),
- lower blood pressure,
- lower postprandial glycaemia and insulinemia,
- facilitate weight control,
- ensure adequate intake of essential nutrients,
- ensure eating pleasure and thus compliance,
- finally reduce the risk for CVD morbidity and mortality.

## 6. Dietary Changes to Improve Dyslipoproteinemia

Traditionally, low-cholesterol (LC) and low-fat-high-carb (LFHC) diets have been recommended to improve dyslipoproteinemia. Yet the value of reducing dietary cholesterol has never been established according to EBM criteria.

The individual response to dietary cholesterol has been extensively studied [7]: whereas about 25% experience a makeable increase of both circulating LDL and HDL cholesterol (hyperresponders), about 75% of the population experiences only a mild increase or no alterations in plasma cholesterol concentrations when challenged with high amounts of dietary cholesterol (normal responders and hyporesponders). Egg intake promotes the formation of large LDL and HDL subclasses, which are less atherogenic [8]. It may even be triggered by the question, whether an individual was breast-fed as an infant, which seems to downregulate cholesterol synthesis.

Although it had been shown very early in the 1950s that dietary cholesterol and eggs are not relevant for serum cholesterol levels [9–11], it took many years to exculpate eggs from being a cause of myocardial infarction (MI) and stroke of CVD [12, 13].

Most cohort studies, among them a recent analysis of NHANES, did not find an association between egg consumption and CVD [14]. Contrasting to that, an analysis from the Physicians' Health Study found an association between egg consumption and total mortality (but not MI and stroke), which was pronounced in diabetic physicians, the reason for which is unclear [15]. Cohort studies are subject to possible confounders. It is not known if there were lifestyle differences between physicians eating less than one egg per week and those eating more than six eggs per week. Interestingly, in the context of a carbohydrate-restricted diet, consumption of three eggs per day compared with no eggs favourably influenced the lipoprotein profile in overweight/obese male subjects [16, 17]. So the effect of egg consumption in individuals with the metabolic syndrome and/or diabetes may be modified by the carbohydrate content of the diet. In conclusion, there is no sufficient evidence to recommend a general restriction of egg consumption, which is still part of many dietary guidelines [12].

**Figure 1:**
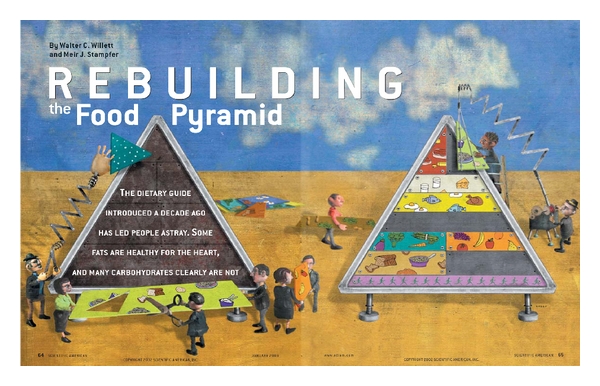
Cover of Willet's 2003 review article [1], with kind permission of Nature Publishing Group.

Further, the much advocated LFHC diets never had been proven to reduce CVD risk, although this still is the assumption of many physicians and nutritionists [18]. As Walter Willett put it in his remarkable 2003 review (Figure 1) in the Scientific American, *“The dietary guide we introduced a decade ago has lead people astray. Some fats are healthy for the heart, and many carbohydrates clearly are not.” *[1].

Besides, the last statement considering carbohydrates made lobbyists of the food industry to accuse Willett of a “political statement”. This finally led to his exclusion from the 2005 committee, which revised the “Dietary Guidelines for Americans”. Food politics always had a great influence on dietary recommendations.

It is noteworthy that before LFHC diets became popular, carbohydrates had been blamed for decades as the main culprit for diabetes, obesity, and coronary heart disease (CHD) [19].

High-quality evidence from meta-analyses of controlled nutrition intervention studies [20, 21] has now contradicted the traditional assumption that LFHC diets are the best recommendation to improve blood lipids. It has been clearly demonstrated that *fat quality* is the most important determinant of changes in blood lipids, not *total fat content* nor the *relative percentage of fat* in a diet [20].

- Exchanging saturated fats (less) for carbohydrates (more) will moderately lower LDL cholesterol but shift LDL subclasses to an unfavourable pattern B (small-dense LDL and higher ApoB). It will lower HDL stronger than LDL and raise triglycerides (TG), thus possibly deteriorating CVD risk, instead of improving it [22–24]. Exchanging carbohydrates (less) for saturated fats (more) induces only a small rise in TC : HDL ratio but will raise LDL size to a more favourable pattern [20, 25]. Of the four major sources of saturated fat (Lauric, myristic, palmitic, and stearic acid) only palmitic acid raises TC : HDL ratio, myristic acid is neutral, and lauric acid and stearic acid lower TC : HDL ratio and may even be beneficial [20, 26].
- Exchanging carbohydrates (less) for mono- or polyunsaturated fats (more) will strongly reduce TC : HDL ratio, lower TG, and favourably influence CVD risk [27].
- Exchanging saturated fats for mono- or polyunsaturated fats will improve TC : HDL ratio, too.
- Only transfatty acids significantly deteriorate TC : HDL ratio if they substitute carbohydrates [20].

Low-fat-high-carb (LFHC) diets are apparently contraproductive and induce unfavourable lipid changes in overweight and obese individuals, who are insulin resistant. The metabolic profile is important for the patient's response to dietary changes: the more insulin resistant, the worse they do with a low-fat-high-carb diet. To the contrary, diets high in monounsaturated fatty acids and lower in carbohydrate content perform much better in T2DM according to a meta-analysis [27–30].

A randomised, controlled, high-quality nutrition intervention study by the group of Ronald Krauss from San Francisco clearly demonstrated the benefit of a low-carb-high-fat diet (LCHF) on blood lipids in overweight men: it compared a “prudent” LFHC diet (54% Carb, 16% Protein, 30% Fat) with a low-fat-high-protein-moderate-carb diet (39% Carb, 29% Protein, 31% Fat), an LCHF diet with predominantly monounsaturated fatty acids (26% Carb, 29% Protein, 46% Fat), and an LCHF diet higher in saturated fat (26% Carb, 29% Protein, 45% Fat). After a 1-week-run-in-phase, where all men ate a control-diet with 54% Carbs, it randomised the participants on the respective four diets for 3 weeks with stable weight (isocaloric). After this period the most favourable changes were seen in the LCHF diet with predominantly monounsaturated fatty acids: TC : HDL ratio was reduced by 13% in the LCHF group, whereas the LFHC group did not show an improvement. Lowering of ApoB was much better in the LCHF groups than in the LFHC group [31]. 

In the following weight-loss period all participants lost weight by calorie restriction on their respective diets. After 9 more weeks, there were improvements in all groups, which clearly separated the effects of diet alone and diet in combination with weight-loss.

This is an important aspect in the discussion, because long-term weight loss, as desirable as it may be, will remain an illusion for many individuals with weight problems. Therefore the more important is the fact that favourable changes in blood lipids are achievable *without weight loss*, if insulin resistant individuals follow an LCHF diet [32].

Thus, isocaloric exchanges of mono/polyunsaturated fatty acids (more) for carbohydrates (less) and/or protein (more) for carbohydrates (less) both improve TC : HDL ratio and LDL : HDL ratio and lower TG levels [20, 31]. The effect of LCHF diets is especially strong in overweight, obese, and/or insulin-resistant individuals.

## 7. Sidestep: Transfatty Acids

A widely neglected, but still very important aspect, is the problem of transfatty acids (TFAs) in fast food, bakery fats, and especially in snack foods [33, 34]. Transfatty acids in human nutrition mainly stem from partially hydrogenated vegetable oils. They raise LDL cholesterol, lower HDL-cholesterol, promote systemic inflammation, and deteriorate insulin sensitivity [35–37]. Unlike Denmark (since 2002), New York (2007), Switzerland (2008), and recently California, there is no ban on the use of TFA nor any obligation to declare its content in foods in Germany or in the European Union. Considering the importance of TFA with strong evidence for a causal link with CVD, there is an urgent need for political action towards a Europeanwide ban.

## 8. Effects of Different Diets on Hypertension

Reducing the dietary sodium content [38, 39] and raising the intake of potassium- and calcium-rich foods [40, 41] (e.g., fruits, vegetables, or dairy products) have been shown to lower blood pressure. The exchange of carbohydrates for either more protein or more unsaturated fatty acids lowered blood pressure and improved lipid profiles in the OmniHEART-Trial [42].

 The DASH Diet is in fact a Mediterranean-style diet with 10 portions of fruits and vegetables per day, more dairy products, low sodium content, and less sugar and starch. Its glycemic load (GL) is reduced in comparison to the average American diet by preferring whole grain products instead of refined carbohydrates.

## 9. Effects of Different Diets on the Risk for T2DM

For quite a while now, the same low-fat-high-carb (LFHC) diet that was proposed for CVD prevention has been recommended in official guidelines to individuals with overweight, MetS, and T2DM. The main focus was lowering of TC and LDL-C, irrespective of the also known effects of such a high glycaemic load on HDL-C and glucose metabolism. A rise in glucose was lowered by medication (oral antidiabetics or insulin). Lately, the negative effects of such an approach, which favours hyperinsulinemia and ß-cell-exhaustion, have been demonstrated [43–45]. In the EPIC study and in a recently published meta-analysis of prospective studies, carbohydrate quantity and quality (GI/GL) were associated with a higher risk for T2DM [43, 46, 47].

Gerald Reaven, the “father” of the concept of insulin resistance and the Metabolic Syndrome, concluded in his 2005 review on dietary approaches in insulin resistance: *“There is considerable evidence that isocaloric diets, low in fat and enriched in CHO, will accentuate the manifestations of the IRS. The more insulin resistant an individual, the greater is the amount of insulin that must be secreted in response to a CHO-enriched diet in order to maintain glucose homeostasis. Thus, the inevitable, and consistently replicated, effect of replacing SF with CHO in insulin-resistant individuals is to increase daylong concentration of glucose or insulin, or both. In addition, this dietary approach has consistently been shown to stimulate hepatic VLDL-TG synthesis and secretion, leading to an increase in concentration of TG-rich lipoproteins, both in the fasting and postprandial states. The increase in the ambient TG-rich lipoproteins seen following low-fat/high-CHO diets has previously been shown to be associated with a decrease in HDL-C concentration, and it appears that such diets will change the LDL subclass pattern to B in 36 of 87 individuals (41%) who had either pattern A or an intermediate pattern at the outset.”* [48].

Interestingly, in the preinsulin era, the dietary approach to treat T2DM was quite different and is experiencing its renaissance in the last years [19].

A recent meta-analysis of 37 prospective observational studies concluded that diets with a high GI or GL independently increased the risk of type 2 diabetes, heart disease, gallbladder disease, breast cancer, and all diseases combined [49]. 

**Figure 2:**
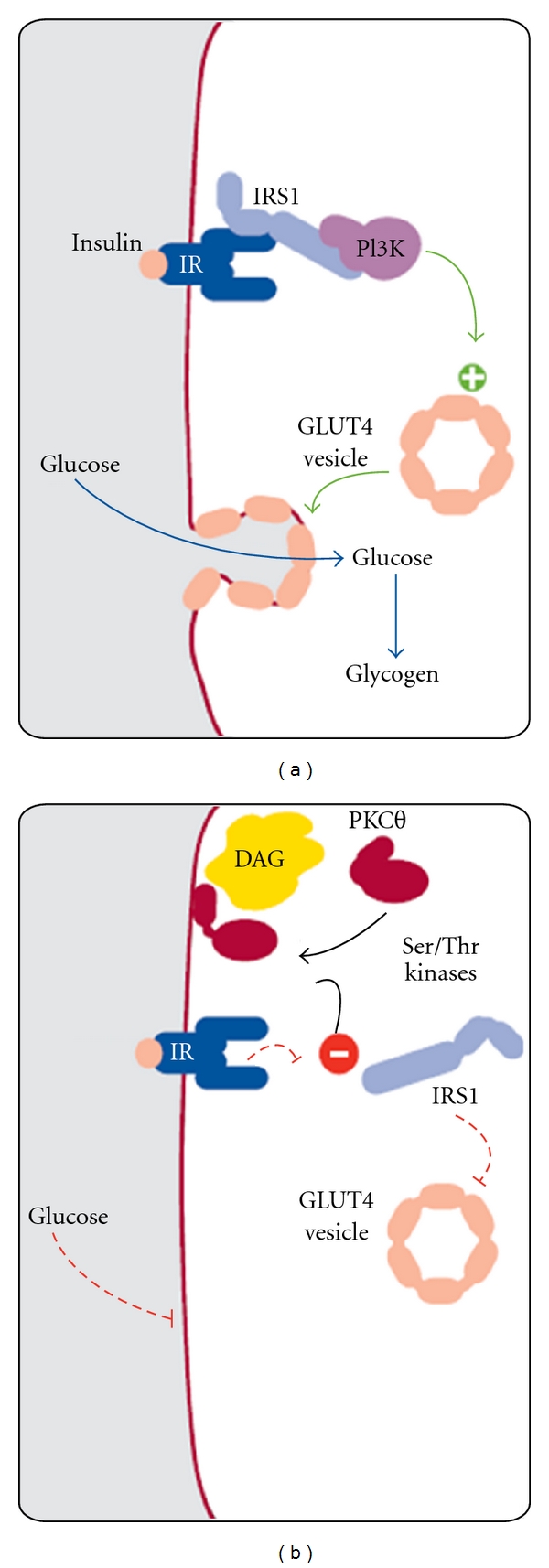
Pathophysiology of muscular insulin resistance: normal (a) and impaired (b) insulin signalling pathway (from [53], reprint with kind permission of Elsevier Limited).

The pathophysiological basis for the detrimental effects of carbohydrates in susceptible individuals has been clarified by the landmark studies of Petersen et al. from Yale University [50–52]. They discovered that an impaired mitochondrial function with a lower rate of conversion of diacyl-glycerol (DAG) to triglycerides (TGs) due to a genetic predisposition will lead to intracellular DAG accumulation, which impairs insulin signalling pathways [53] (Figure 2).

In combination with physical inactivity especially this causes muscular insulin resistance with reduced glucose uptake. After a high carbohydrate meal under these conditions, glucose will be redirected to the liver, where it induces de novo lipogenesis, fat storage in the liver, and finally hepatic insulin resistance with visceral obesity and all its known consequences. From the same amount of ingested carbohydrates, lean, but insulin resistant students will have a more than doubled de novo lipogenesis in liver as their insulin-sensitive controls. One short bout of physical activity can reverse this effect, underlining the importance of a lifestyle approach combining dietary with physical activity recommendations in insulin resistant subjects [54].

Also, genetic deficits in insulin secretion may play a role in the development of T2DM [55]. The higher the glycaemic load of the diet, the more difficult it is for individuals with this frequent genetic mutation, to achieve glucose homeostasis. Besides, muscular activity can reverse the negative effects of this genetic deficiency, too, as has been shown in the American Diabetes Prevention Study [55]. 

On the other hand, controlled nutrition intervention studies have validated the influence of glycaemic load on postprandial glycaemia and have shown a benefit of carbohydrate-restricted diets with a higher content of protein and fat in prediabetic subjects as well as in diabetics [56–65]. 

Considering this, it is not a surprise that the latest publication from the Kuopio study published in February 2011 showed the GI/GL to be associated with a significant increase in the risk for acute myocardial infarction in overweight and/or physically inactive men [66].

## 10. Dietary Changes and Weight Loss

Controlled dietary intervention studies have demonstrated that low-carb-high-fat (LCHF) diets or low-glycaemic-index (LOGI) diets in comparison to low-fat-high-carb (LFHC) diets double the weight loss achieved over 6 months [67–73]. Also Low-Glycemic Load Diets (LOGI) have shown superior weight loss [74–76]. In insulin-resistant subjects especially, there is a better weight loss, when carbohydrates and/or glycaemic index are reduced. The myth that fat content of a diet is the main culprit for weight gain and obesity has been contradicted by these studies.

Interestingly, most studies tested *calorie-restricted* low-fat diets against *ad libitum* low-carb diets, and still low-carb performed better. The explanation is that low-carb but high-protein and high-fat diets promote better satiety than the traditional low-fat, low-protein, high-carb diets.

And furthermore, the energy density of a diet, which is responsible for the initial satiating effect, depends more on the *water and fibre content* of a diet than on the *relative* fat content. A “high-fat” diet with a *relative* fat content of 40–50% en but with plenty of fruits and vegetables can still be “low-caloric”, because of a low energy density of the combined meal. This common misunderstanding of the term “high-fat” should be clarified at this point.

As the compliance with all dietary changes tends to degrade with time, regain of weight is initiated typically after 6–12 months. But still after 12 to 24 months there is a trend for higher weight loss on LCHF diets [73].

A recent meta-analysis of weight loss diets by the Cochrane Collaboration leads the authors to the following conclusions: *“Overweight or obese people on low glycaemic index diets lost more weight than those on high glycaemic index diets or conventional energy restricted weight loss diets, with the change in body mass, total fat mass and body mass index all significantly decreasing after the low glycaemic index diet compared to the comparison diet. It may be easier to adhere to a low glycaemic index diet than a conventional weight loss diet, since there is less need to restrict the intake of food as long as low glycaemic index carbohydrates are predominantly consumed. In studies comparing ad libitum reduced glycaemic index or load diets to conventional restricted low fat diets, even though participants could eat as much as desired on the low glycaemic index or load diets, they fared as well, or better, in the outcomes than those on the comparison diet. Hence, lowering the glycaemic index of foods in the diet appears to be an effective method of losing weight, particularly for the obese.”* [74].

## 11. Conclusion and Practical Approach

The aforementioned cited evidence convincingly argues for a change in dietary recommendations towards the prevention of CHD. In those patients with preponderance to overweight and diabetes especially, and in those who are most of the day physically inactive, reducing carbohydrates (low GL) is beneficial, if carbs are exchanged for good fats and/or protein.

Renowned institutions like the Joslin Diabetes Center already have revised their nutrition guidelines (Clinical nutrition guideline for overweight and obese adults with type 2 diabetes, prediabetes, or those at high risk for developing type 2 diabetes).

As any dietary strategy should be evidence-based, effective, and—in a real-life setting—practical from the patients' point of view, it has to be palatable, too.

**Figure 3:**
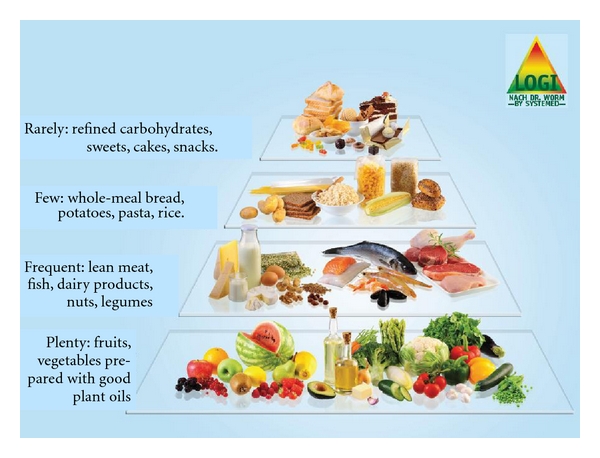
The “LOGI pyramid” according to Dr. Nicolai Worm, revised version 08/2009.

One of most successful approaches into this direction is the LOGI concept created by David Ludwig, Harvard University, which was made popular in the German version by Dr. Nicolai Worm (Figure 3).

LOGI is “mediterranian-style low-carb” and combines practicability and palatability: it is based on an abundance of plant-based foods (fruits, low-starch vegetables, and salads), combined with “good fats” (monounsaturated and omega-3-polyunsaturated, olive oil, canola oil, nuts, seeds, fish), more protein (low-fat dairy products, legumes, fish, poultry), and only small amounts of the classical carbohydrate-rich foods like pasta, whole-meal-bread, rice, and potatoes.

Breakfast cereals, snacks, sweets, and sugar-sweetened beverages should be strictly limited. From the two basic levels (fruits/vegetables/salads/good fats, and protein sources), patients can eat ad libitum, which makes compliance very good with LOGI. There is a wealth of literature about LOGI with excellent cooking book for patients, containing easy-to-prepare recipes with calculations for energy density, carb/fat/protein content, and nutritional values (see http://www.logi-methode.de/).

## 12. Proposed Practical Dietary Recommendations for the Prevention of CVD

- Change *fat quality* to improve dyslipoproteinemia:
  1. good fats: olive oil, canola/rapeseed oil, nut oils, nuts, seeds, and fish with omega-3-fatty acids,
  2. neutral fats: saturated fats in milk products and cheese,
  3. bad fats: fat in processed meat, sausages, transfatty acids in bakery products, cookies, sweets, and so forth.
- Eat plenty high-volume-low-calorie foods: vegetables, salads, and fruits.
- Reduce total carb intake, prefer low-glycaemic index foods, and reduce glycaemic load to lower fasting and postprandial glucose and insulin levels (the more insulin resistant, the stronger this recommendation is to be followed!):
  1. good carbs: nonstarchy vegetables, salads, legumes, most fruits, and whole and minimally processed grains,
  2. if you eat carbs, eat few and prefer whole grain bread, brown rice, and pasta,
  3. carbs to avoid: low-fibre cereals, sugars, HFCS (high-fructose-corn-sirup) sweetened softdrinks, snacks, pizza, fries, baked potatoes, sweets, white and brown bread, and all foods containing refined starch.
- Eat more protein for better and longer lasting satiety:
  1. good protein sources: fish, poultry, low-fat dairy products, and lean-cut meat.
  2. protein sources to avoid: processed red meat and sausages.
- Moderate alcohol consumption:
  1. regular small amounts of alcohol (preferably wine) can be part of a healthy diet (10–20 g per day),
  2. if individuals prefer to drink small amounts of wine on a regular basis, it is OK,
  3. excessive drinking is detrimental to health,
  4. nobody should start drinking alcohol for preventive reasons,
  5. alcohol consumption should always be part of a culture of healthy good cooking (Mediterranean style).
- Physical activity recommendations: 30 min daily of moderate physical activity is the minimum amount necessary for health (and should be part of the nutrition guideline, too).
